# A generic standard additions based method to determine endogenous analyte concentrations by immunoassays to overcome complex biological matrix interference

**DOI:** 10.1038/s41598-017-17823-y

**Published:** 2017-12-13

**Authors:** Susan Pang, Simon Cowen

**Affiliations:** 0000 0004 0556 5940 grid.410519.8 LGC, Queens Road, Teddington, UK

## Abstract

We describe a novel generic method to derive the unknown endogenous concentrations of analyte within complex biological matrices (e.g. serum or plasma) based upon the relationship between the immunoassay signal response of a biological test sample spiked with known analyte concentrations and the log transformed estimated total concentration. If the estimated total analyte concentration is correct, a portion of the sigmoid on a log-log plot is very close to linear, allowing the unknown endogenous concentration to be estimated using a numerical method. This approach obviates conventional relative quantification using an internal standard curve and need for calibrant diluent, and takes into account the individual matrix interference on the immunoassay by spiking the test sample itself. This technique is based on standard additions for chemical analytes. Unknown endogenous analyte concentrations within even 2-fold diluted human plasma may be determined reliably using as few as four reaction wells.

## Introduction

Standard curves are used in immunoassays to interpolate unknown concentrations of the analyte of interest within biological test samples by relative quantification using the observed signal response. Typically, in addition to a zero calibrator, a seven-point calibration curve is constructed by spiking known analyte concentrations into a diluent without endogenous analyte that emulates the matrix complexity^1^. Interferences in quantitative immunoassays are well documented^2,3^. When matrix effects are neutralised the calibration curve overlays the signal response curve of the spiked biological test sample when evaluating the total analyte concentration. High fold-dilutions of the test sample may nullify matrix interference if the immunoassay affords sufficient sensitivity. However, many proteins of clinical interest (e.g. disease-state biomarkers) are rare plasma proteins, and their detection is hampered by the huge dynamic range of proteins extending over 10 orders of magnitude^4^. Hence, substantial sample dilution may not always be feasible. With some immunoassays differential matrix effects may even be exhibited among distinct plasma samples due to dietary intake^5^. Limiting sample dilution to preserve the analyte concentration within the assay detection range while accounting for matrix interference on a case-by-case scenario would be desirable.

**Figure 1:**
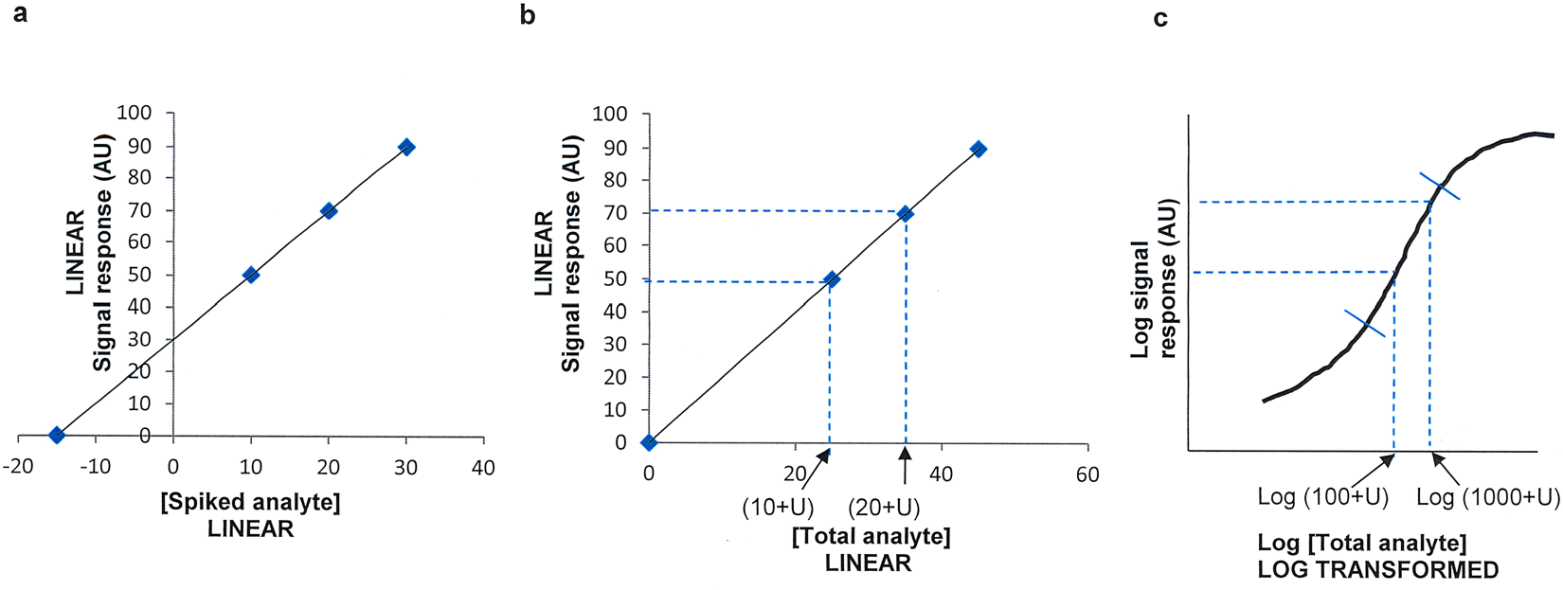
Illustrations depicting conventional standard additions for chemical analytes and biological targets by immunoassay. Figure 1a: A typical plot for illustrating the method of standard additions for chemical analysis. The intercept at -15 indicates the endogenous concentration of the chemical analyte in this example is 15 units. Figure 1b: An alternative view of Supplementary Fig. 1a to show the relationship if the total analyte (not just spike concentration) is plotted on the x-axis, where U denotes the unknown endogenous chemical analyte concentration. If a linear change in the concentration of a chemical analyte exhibits a linear change in response, the gradient can be resolved for the line of fit, and the unknown endogenous concentration can then be determined; i.e. using the equation for a straight line: y = mx + c, where y is the response, x = concentration and c = intercept. Therefore, with c = 0, 70–50 = m(20 + U−10−U) gives a gradient of m = 2. Therefore if y = 2x, then 50 = 2(10 + U), and U = 15 units. Figure 1c: The analogous plot for the signal response curve for an immunoassay of a biological analyte where part of the log transformed response and log transformed total analyte concentration give rise to a linear correlation which cannot be solved in the same manner as shown as for a chemical analyte (as shown in Fig. 1b) in spite of a linear correlation within the sigmoid, due to the log transformation of the concentrations on the x-axis.

We propose a novel method to determine the unknown endogenous analyte concentration within biological samples by directly spiking the samples with known concentrations of standards, and compare the findings with data derived using the conventional method of relative quantification where values are interpolated from a standard curve. Our approach is based on the method of standard additions, a quantitative approach used in analytical chemistry where standards are spiked directly into the test sample to eliminate matrix effects when determining the endogenous analyte concentration by measuring the linear signal response correlating to a linear change in concentration (Fig. 1a,b)^6^. Conventional standard additions cannot be implemented for immunoassays as the signal response from the antibody-antigen interaction is generally sigmoidal when both the concentration and response are log transformed due to cooperative binding (Fig. 1c). The 4-parameter logistic (4-PL) model is generally favoured for fitting sigmoidal calibration curves^7^. Simpler models such as the “log-log linear” model may be used to selectively fit the linear portion of the sigmoid if the linear range (on a log-log plot) accommodates the desired detection range^8^. Hence, if a test sample is spiked with standards, and the estimate for the unknown endogenous analyte concentration is correct, then the correlation between the logarithm of the total analyte concentration and the logarithm of the response should produce a sigmoidal curve that includes an approximately linear region. Working within this linear range means that the relationship between total concentration and signal response can be treated as a simple linear regression.

Here we report a method utilising linear regression to obtain an estimate of the endogenous analyte concentration *U* analogous to conventional standard additions. The basis of the method is as follows: an initial estimate of *U* is made, and the logarithm of the putative total concentration calculated. If the spiking levels are such that the maximum linear portion of the sigmoid on a log-log plot is correctly identified and included in the linear correlation, and the estimated total analyte concentration is correct, then a linear relationship exists between the log response and the log total concentration (Supplementary Fig. 1); but is otherwise non-linear. This provides a means to estimate *U*: by finding the estimated value which produces the most linear relationship. Essentially, this amounts to changing the estimate until the relationship is closest to linear, and requires a suitable numerical algorithm and a criterion for judging linearity. An implementation using the Microsoft Excel Solver is described in the Methods section.

Nonlinear regression can also be used for problems of this type^9^, but are difficult to employ without specialist support. Our method overcomes this, and can deliver good results with as few as four spike levels if the repeatability precision is sufficiently high.

Our method accommodates non-linearity, which traditional standard additions cannot generally achieve. It may be implemented for sandwich and competitive immunoassays, though the latter is more prone to matrix effects given the use of a single antibody. We have illustrated the approach via the detection of endogenous cortisol in human serum (adopting a competitive assay using certified reference materials (CRMs)), and amyloid beta (Aβ) peptides in human plasma (via sandwich assays) using the Meso Scale Discovery (MSD) SECTOR Imager 6000 instrument.

## Results

### Derivation Of Endogenous Analyte Concentrations By Linear Regression Illustrated By Competitive Cortisol Immunoassays In Human Serum

**Figure 2:**
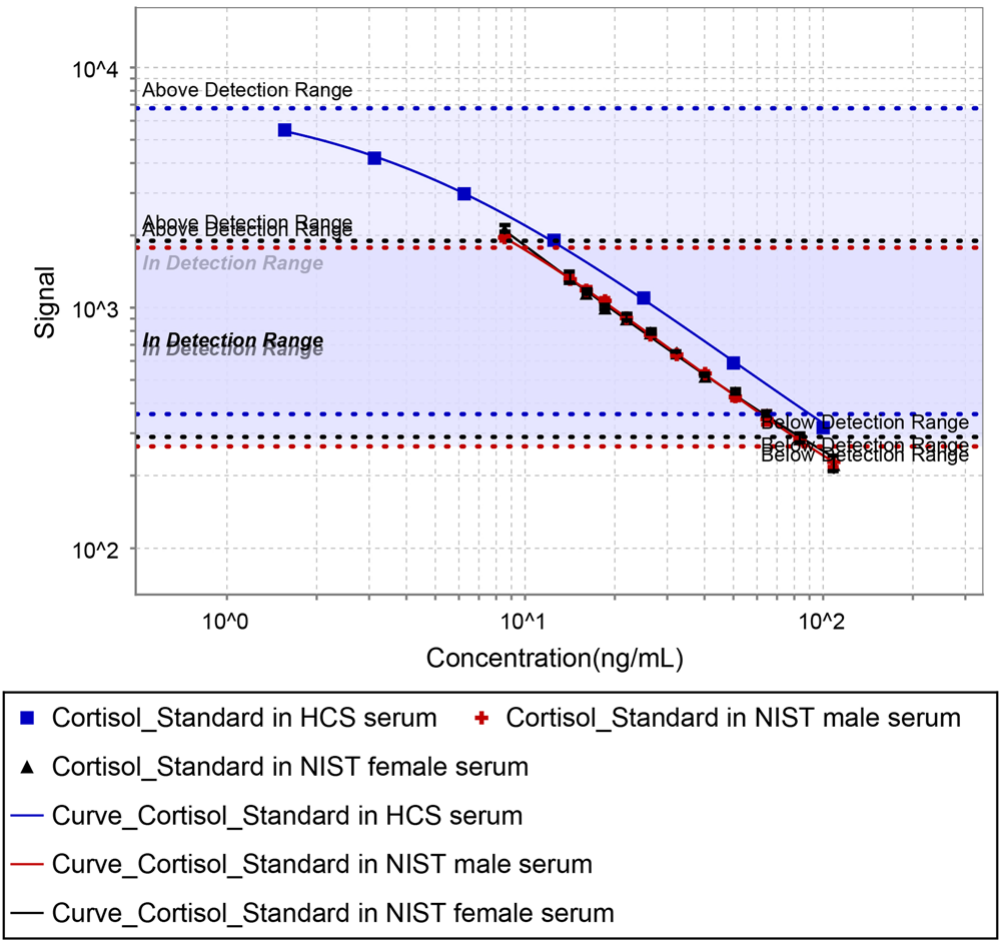
Cortisol standard curves in diluent and test plasma. The 4 parameter logistic fit of the cortisol standard curve in a background of (blue line) 12-fold diluted heavy charcoal stripped (HCS) serum, (red line) 12-fold diluted NIST male serum (NIST 971) and (black line) 12-diluted NIST female serum (NIST 971), using NIST cortisol CRM 921 as the calibrants.

Standard curves were constructed from signal response data derived from human serum samples spiked with known concentrations of cortisol standards, taking into account the endogenous and exogenous cortisol concentrations (both known via the use of CRMs from the National Institute of Standards and Technology (NIST)), using 4-PL curve fitting (MSD Discovery Workbench v3.0 software). The curves for the spiked male and female NIST sera do not overlay with the calibration curve in a diluent of heavy (4x) charcoal-stripped serum (Fig. 2). Although the spiked sample curves are parallel to the conventional calibration curve comprising of cortisol standards in charcoal stripped serum, the shift of the calibration curve to the right of the test sample curves results in overestimations of test analyte concentrations interpolated from the calibration curve. Despite overestimation in analyte recoveries (i.e. 138–151% and 137–148%, respectively, for male and female NIST sera; Table 1) when the endogenous concentration of cortisol is interpolated from the standard curve, recovery is not concentration-dependent and the recovery range is narrow (≤13%) given that the acceptance range for the recovery is usually 80–120% which extends over 40%. Charcoal extraction is likely to remove more constituents than just hormones, reducing the serum complexity compared with unprocessed test serum, thus resulting in overestimation when using conventional relative quantification.

**Table 1:** A summary of the signal outputs and backfitted concentrations and recoveries of cortisol.

Sample Group	Net plasma dilution after spike addition	Conc (ng/mL)	Mean Signal	CV	Calc. Conc. Mean	Calc. Conc. SD	Calc. Conc. CV	Predicted total cortisol for neat sample (ng/mL)	% Recovery
Standard		100	316	1.1	105.3	1.4	1.3		105.3
Standard		50	588	0.8	51.0	0.5	1.0		101.9
Standard		25	1097	1.1	24.5	0.3	1.3		98.2
Standard		12.5	1908	1.7	12.2	0.3	2.3		97.4
Standard		6.25	2978	1.7	6.3	0.2	2.8		100.5
Standard		3.125	4191	1.2	3.3	0.1	2.8		104.2
Standard		1.563	5491	1.9	1.5	0.1	7.1		95.8
Standard		0	7421	3.0	0.1	NaN	NaN		
M1	12		228	5.4	1896.5	134.1	7.1	1302.5	145.6
M2	12		283	1.9	1448.6	34.4	2.4	1002.5	144.5
M3	12		343	5.7	1149.8	80.9	7.0	777.5	147.9
M4	12		426	4.6	890.2	49.2	5.5	608.7	146.2
M5	12		532	1.9	687.7	15.3	2.2	482.2	142.6
M6	12		638	2.1	556.9	13.2	2.4	387.2	143.8
M7	12		770	2.4	447.9	12.5	2.8	316.0	141.7
M8	12		905	1.9	370.6	8.2	2.2	262.6	141.1
M9	12		1068	2.4	304.4	8.8	2.9	222.6	136.8
M10	12		1181	2.7	269.5	8.8	3.3	192.6	140.0
M11	12		1313	2.0	236.6	5.8	2.5	170.0	139.2
M12	12		1956	3.0	141.4	5.8	4.1	102.5	137.9
F1	12		222	7.7	1964.7	201.2	10.2	1302.5	150.8
F2	12		285	4.5	1438.5	80.7	5.6	1002.5	143.5
F3	12		358	1.7	1089.9	21.8	2.0	777.5	140.2
F4	12		443	1.9	850.6	19.0	2.2	608.7	139.7
F5	12		519	0.9	707.5	7.3	1.0	482.2	146.7
F6	12		649	0.5	546.1	3.0	0.5	387.2	141.0
F7	12		786	1.6	437.2	8.2	1.9	316.0	138.3
F8	12		899	3.2	373.5	13.9	3.7	262.6	142.2
F9	12		997	1.9	330.2	7.5	2.3	222.6	148.3
F10	12		1149	2.0	278.6	6.6	2.4	192.6	144.7
F11	12		1323	4.7	234.7	14.1	6.0	170.0	138.0
F12	12		2103	3.1	127.9	5.6	4.4	86.4	148.0

Data are shown for male NIST sera (M1−12) and female NIST sera (F1−12) ± exogenous cortisol CRM spikes obtained by conventional relative quantification from the 4-PL fit curve using the default MSD software.

However, when using linear regression to correlate the log transformed signal output and log transformed estimated total cortisol concentrations of the 11 spiked and one unspiked sample for each test sera, the estimated concentrations of the 12-fold diluted NIST male and female sera were 9.4 ng/mL and 7.7 ng/mL, respectively (Supplementary Fig. 2). This equates to cortisol recoveries of 107% and 112%, for male and female NIST sera, respectively, thus minimising the extent of overestimation that is observed with relative quantification.

Spiking every test sample with 11 distinct calibrants is impractical, due to greater expense, sample and reagent consumption. Hence we explored if fewer spiked concentrations and replicate loadings may be used to derive reasonable estimations of endogenous cortisol concentrations.

Linear regression derived endogenous concentrations of cortisol were obtained using different combinations of fewer data points (i.e. mean values of triplicate determinations of signal outputs); Table 2. The robustness of this method depends on a sufficient number of spikes to cover the optimum linear correlation, and the closeness of the linear approximation in the working range. The ideal scenario entailed four data points to encompass the maximum linear range constituting the longest sufficiently linear portion of the curve with even coverage of points in a log space, i.e. spike solutions 1 and 12 in combination with either spikes 4 and 8 or spikes 5 and 9, as the uncertainty in the fitted line is reduced^10^. With these combinations the cortisol recovery was 95–116%. Attempts to use a narrower range with four points encompassing a narrower portion of the linear range, e.g. spikes 5–8 may lead to severely imprecise estimates, as the line and fitted value becomes increasingly poorly defined as the range narrows^10^.

**Table 2:** Linear regression derived cortisol concentrations and recoveries determined using different combinations of the 11-spike concentrations of the male and female test sera.

Points	Net plasma dilution after spike addition	NIST Serum	Spike solutions used in linear regression method	Linear regression derived concentration of Cortisol in diluted serum (ng/mL)	Linear regression derived concentration of Cortisol in neat serum (ng/mL)	Residual sum of squares	% Recovery (based on LC-MS assigned cortisol within the CRMs from NIST)
12	12	Male	1–12	9.4	112.8	0.00017	110.1
11	12	Male	1–11	7.6	91.1	0.000164	88.9
11	12	Male	2–12	10.0	120.0	6.44xE−05	117.1
10	12	Male	2–11	8.4	101.4	0.001299	98.9
9	12	Male	2–10	7.6	90.8	0.000198	88.6
9	12	Male	3–11	10.2	122.8	1.98xE−05	119.8
8	12	Male	3–10	9.6	114.9	0.00051	112.2
5	12	Male	1,4,7,10,12	9.2	110.1	0.000891	107.4
4	12	Male	1,4,8,11	7.2	86.6	0.000392	84.5
4	12	Male	1,4,8,12	9.0	108.0	0.000735	105.4
4	12	Male	3,6,8,12	10.3	124.2	4.76xE−05	121.2
4	12	Male	5,6,7,8	5.1	60.7	4.84xE−05	59.2
4	12	Male	6,7,8,9	4.5	54.4	5.22xE−05	53.1
4	12	Male	1,5,9,12	9.9	118.6	0.000745	115.7
4	12	Male	1,4,7,10	6.9	83.3	0.000356	81.2
12	12	Female	1–12	7.7	92.8	0.004967	107.3
11	12	Female	1–11	9.6	115.6	0.004041	133.8
11	12	Female	2–12	7.2	87.0	0.003838	100.6
10	12	Female	2–11	8.5	102.1	0.003577	118.2
9	12	Female	2–10	10.9	130.8	0.002512	151.3
9	12	Female	3–11	7.9	94.4	0.003481	109.3
8	12	Female	3–10	10.9	130.8	0.002512	151.3
5	12	Female	1,4,7,10,12	8.3	99.3	0.002196	114.9
4	12	Female	1,4,8,11	9.7	115.9	0.000346	134.1
4	12	Female	1,4,8,12	8.3	99.6	0.000539	115.3
4	12	Female	3,6,8,12	7.5	89.5	3.42xE−05	103.6
4	12	Female	5,6,7,8	101.9	1222.3	5.71xE−05	*N/A
4	12	Female	6,7,8,9	130041.0	1560489.0	2.34xE−05	*N/A
4	12	Female	1,5,9,12	6.9	82.3	0.000242	95.2
4	12	Female	1,4,7,10	13.8	165.5	6.54xE−05	191.5

The residual sum of squares is the parameter minimised in a least squares linear regression, and is the sum of the squared difference between observed and fitted values.

*N/A denotes that the recovery determined by this approach was ≥200%, and that the combination of data points used to derive the endogenous analyte concentration was inappropriate.

### Reproducibility Of Linear Regression Derived Concentrations

While validating the total cortisol assay, each NIST sera was spiked separately with three distinct concentrations of standards to emulate the upper physiological range of total cortisol when using a 12-fold sample dilution. These samples, along with the unspiked sera were routinely used to assess the reproducibility of cortisol recovery, and the signal output for each replicate loading of sample from all three experiments is shown in Supplementary Table 1. Albeit this combination of spike concentrations did not constitute the optimum maximum linear range as the net concentration of the highest spike was 50 ng/mL, not 100 ng/mL, the reproducibility of the standard additions derived endogenous cortisol concentration *U* was investigated using these replicate experiments. With the mean values from triplicate determinations of signal outputs from three separate experiments, *U* for the male and female sera were 159 ± 29 ng/mL (inter-assay CV = 18%) and 129 ± 14 ng/mL (inter-assay CV = 11%), respectively (Supplementary Table 2). While this particular combination of net spike concentrations (of 0, 12.5, 25, and 50 ng/mL exogenous cortisol) overestimates endogenous cortisol, it demonstrates good reproducibility of the technique using only four distinct spike concentrations. Overestimated endogenous cortisol was also derived when using mean signal output data from the four closest net spike concentrations of 0, 13.35, 23.73, and 56.25 ng/mL cortisol from the experiment with 11 spike concentrations, but this bias is reduced when four points are selected to encompass the maximum linear range as aforementioned, with recoveries of 95–116% (Table 2). When conventional relative quantification was used to determine the endogenous cortisol within male and female NIST sera with these three replicate experiments, the concentrations were 121 ± 6 ng/mL (inter-assay CV = 5%; recovery: 119%) and 108 ± 1 ng/mL (inter-assay CV = 1%; recovery: 125%) as shown in Supplementary Table 2. With this competitive cortisol immunoassay, the linear portion of the log-log plot of the concentration versus signal output is broad, extending well beyond upper physiological levels of total cortisol. This offers scope to detect large increases in cortisol due to high intensity exercise^11^ or hospitalisation of patients^12^. For such applications, hand-held devices affording portable testing would be more desirable than laboratory-based tests. Point-of-care devices often have fewer reaction chambers to achieve portability. Hence, single determinations of signal output for each test sera spiked at 4 distinct concentrations (including a zero spike) were also evaluated to ascertain the scope of deriving the endogenous cortisol concentration from only four reaction wells of a microtiter plate. To gain an indication of precision using single replicates, the mean concentration of cortisol within NIST male serum were determined using only the first replicate at each level, then the second, and then the third. The mean value is 152 ± 46 ng/mL, whereas the value would be 149 ng/mL if the mean of triplicate determinations were used for the same single experiment. Following the same strategy for the female serum data, the average of the linear correlation derived endogenous cortisol from the same three combinations of single determinations is 113 ± 4 ng/mL, and the value is 113 ng/mL if triplicate determinations for each point were first averaged prior to applying linear regression to the spiked test samples on the basis of their total cortisol (known spike and unknown endogenous) concentrations and the corresponding mean signal output (Supplementary Table 2). Albeit the CV increases to 30% when using a single determination, fold-changes in cortisol levels are anticipated in athletes when training. Therefore, linear regression derived endogenous concentrations from single determinations (using only four reaction wells) and the mean of triplicate determinations of the signal output data may produce comparable results.

### Derivation Of Endogenous Analyte Concentrations Using The Proposed Standard Additions Method Illustrated By Sandwich Aβ Peptide Immunoassays In Human Plasma

**Figure 3:**
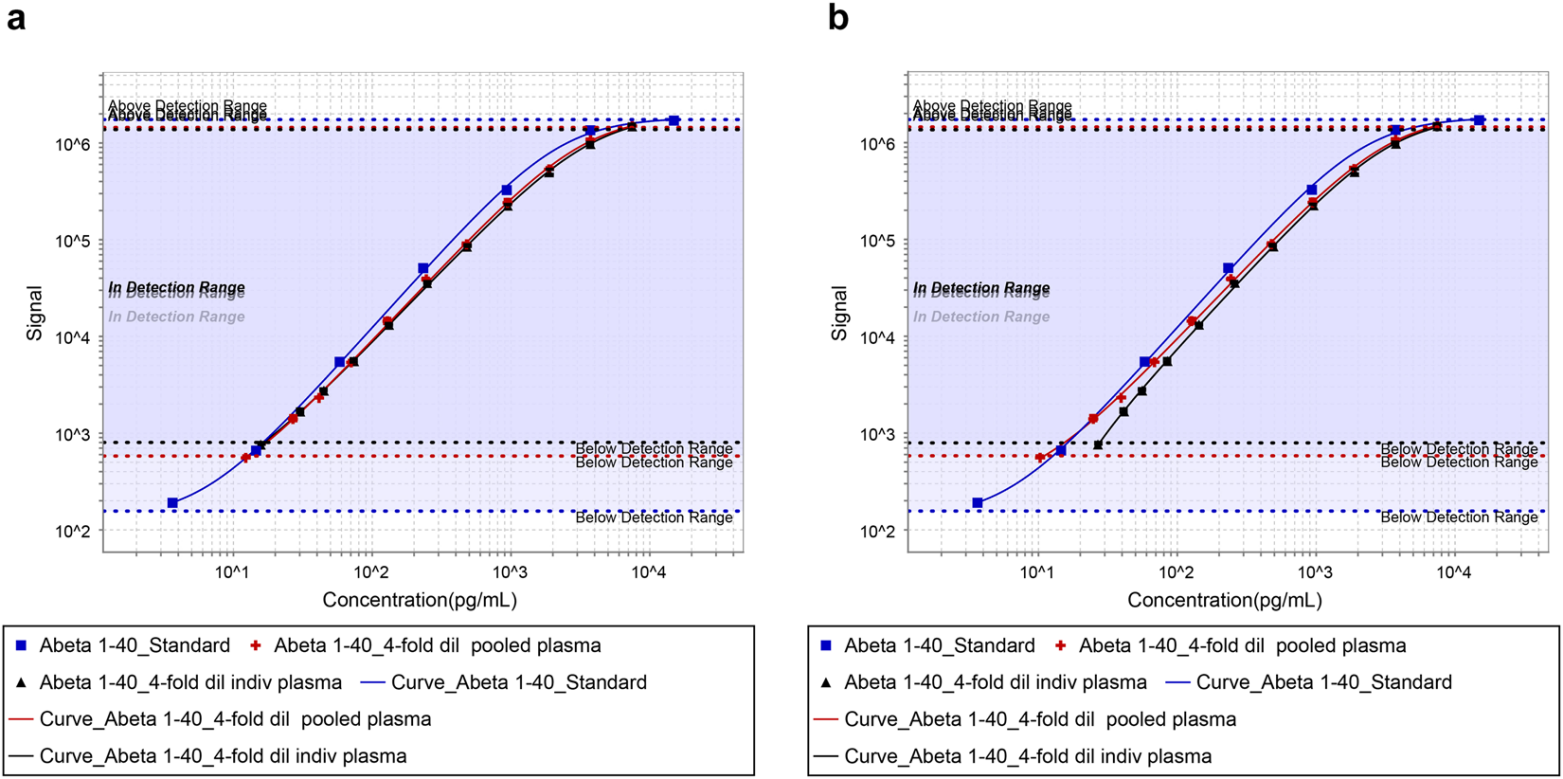
Comparison of signal response curves incorporating endogenous Aβ40 concentrations determined by relative quantification and linear regression. The signal response for the conventional calibration curve (blue curve) and the spiked plasma curves: i.e. 4-fold diluted plasma samples (both the pooled plasma (red curve) as well as the individual plasma A07 (black curve)) were plotted against the total analyte concentration incorporating the endogenous Aβ40 concentration derived (**a**) by relative quantification by interpolating from the standard curve using the MSD software, and (**b**) by using linear regression.

Our novel method was also applied to detect Aβ peptides; Alzheimer’s disease biomarkers^13^, using MSD V-PLEX Aβ Peptide Panel 1 (4G8) kits compatible with human cerebrospinal fluid (CSF) or (≥ 4-fold) diluted mouse plasma. Although Diluent 35 was formulated to emulate CSF it is used for both matrices as the kit calibrant diluent. Aβ40 peptide concentrations within two distinct human plasma samples (one female pooled sample and one individual female plasma termed A07), at two different dilution factors (4-fold and 12-fold) were compared using the conventional method relative quantification as well as our proposed standard additions method. When endogenous Aβ40 derived by relative quantification are incorporated into the total Aβ40 concentration within the diluted plasma samples, the signal response curves of the spiked plasma curves do not overlay with the calibration curve in Diluent 35 (Fig. 3a). This indicates differential matrix interference for the Aβ40 assay within Diluent 35 compared with plasma (at both dilutions), with greater bias at higher concentrations when using relative quantification to interpolate the unknown concentrations on the basis of the observed signal outputs of the test samples. The gradients of the linear portions of all curves (on log-log plots) in diluted plasma are similar, regardless of dilution factor or plasma sample. While the lower tails of the spiked plasma curves are anchored to intercept with the calibration curve at the concentration of the unspiked plasma sample, the spike plasma curves are only correct if the interpolated concentrations of the unspiked plasma samples are accurate by relative quantification. Any deviations between the actual and observed concentrations for the unspiked plasma samples will have a more profound effect in changing the shape of the curve at the lower concentrations due to log transformation. The upper end of the curve for a sandwich assay is more resilient to small changes in the unknown endogenous concentration of the analyte, especially if physiological levels are relatively low compared to a high spike (and subsequently high total) concentration incorporated within the upper working range of the assay, which is the case with this assay. The linear regression data analyses performed to ascertain the endogenous Aβ40 within the plasma samples at both dilution factors are shown in Supplementary Table 3. The linear regression data encompassing the optimum four data points for maximum linearity were derived and compared with the peptide concentrations derived by conventional relative quantification are shown in Table 3. Interpolation of data from the internal standard curve by relative quantification resulted in a slight overestimation in Aβ40 in pooled plasma, and underestimation of Aβ40 in the individual plasma by almost 2-fold, when compared with the standard additions method. With both methods, it is evident that the concentration of Aβ40 is higher in the individual female plasma (A07) than within the pooled plasma. The signal outputs for Aβ40 in both samples at both dilutions were within the working range of the conventional immunoassay utilising relative quantification. The two methods of deriving the endogenous Aβ40 concentrations (relative quantification and the standard additions approach) exhibited similar linearity of dilution with the individual plasma A07. However, for the pooled plasma, the linearity of dilution was improved using the standard additions method to derive the endogenous Aβ40 concentration rather than using conventional relative quantification (Table 3).

**Table 3:** Comparison of endogenous Aβ40 concentration data derived by conventional relative quantification of the unspiked test samples and the standard addition approach using linear regression to correlate the signal output with the total analyte concentration of spiked test samples.

Analyte and test sample	MSD software derived conventional relative quantification via interpolation from a standard curve			Standard additions approach using linear regression and minimal residual sum of squares to ascertain the endogenous analyte concentration		
	Net plasma dilution after spike addition	[Aβ40] in equivalent neat plasma (pg/mL)	**Mean [Aβ40] from two dilution factors**	Net plasma dilution after spike addition	[Aβ40] in equivalent neat plasma (pg/mL)	**Mean [Aβ40] from two dilution factors**
Aβ40 in pooled plasma	4-fold	49.01	**45.7 ± 4.6 (CV: 10.1%)**	4-fold	41.09	**42.8 ± 2.4 (CV: 5.7%)**
	12-fold	42.45		12-fold	44.53	
Aβ40 in individual plasma A07	4-fold	62.85	**60.2 ± 3.73 (CV: 6.2%)**	4-fold	107.18	**112.4 ± 7.4 (CV: 6.6%)**
	12-fold	57.58		12-fold	117.68	

A summary of endogenous Aβ40 concentrations within the pooled female human plasma and the individual female human plasma sample termed A07, determined using conventional relative quantification from a 4-PL fit curve and the novel method of linear regression based on minimising the residual sum of squares to assess concordance when the dilution factor is corrected.

When the gradient of the linear portion of the curve is steeper for the calibration curve than for the spiked plasma curves there are three possible scenarios for the curves to reside relative to each other if the calibration diluent is a simpler solution than the biological test sample (Supplementary Fig. 3a-c). With the first scenario, the spiked plasma curve starts from the datum point for the unspiked plasma localised on the calibration curve with the two curves diverging (Supplementary Fig. 3a). This is the least likely scenario as it would be serendipitous for a test sample that is known to exhibit differential matrix interference from the assay in calibrant diluent (as evidenced by their distinct gradients) to possess the one and only Aβ40 concentration where relative quantification provides an accurate value in spite of matrix differences. With the second scenario, the two curves intercept at a low concentration leading to a slight overestimation of Aβ40 in plasma when the concentration is lower than the point of intercept, and underestimation if the concentration is higher than the intercept, in a concentration-dependent manner (Supplementary Fig. 3b). The third possibility is for the spiked plasma curve to reside fully to the right of the calibration curve such that all the interpolated concentrations of Aβ40 within matrix will be underestimated (Supplementary Fig. 3c). If the standard additions derived endogenous concentrations of Aβ40 peptide are taken into consideration on the plots of the spiked plasma samples in comparison with the calibration curve, the pooled plasma fits the second scenario, whereas the matrix effects exhibited by the individual plasma (A07) resembles the third model (Supplementary Fig. 3b). The gradients of the calibration curves are steeper than the spiked plasma gradients regardless of the method used to derive the endogenous Aβ40, and hence it is evident that the matrix interference for Aβ40 detection in Diluent 35 differs and diluted plasma differ. The only way to account for the unique matrix interference exhibited by each individual biological sample is to spike the test sample with standards. The pooled female plasma was analysed further in a separate experiment utilising 2-fold and 4-fold diluted plasma (Supplementary Table 4) to ascertain the reproducibility of deriving the endogenous analyte concentrations of amyloid peptides by standard additions. Correcting for sample dilution, the concentrations of Aβ40 derived using 2-fold and 4-fold diluted pooled plasma were both 40.2 pg/mL; these values are consistent with the mean value of Aβ40 from the two dilutions of plasma within the first experiment comprising of 42.8 pg/mL. Using 2-fold diluted plasma also afforded detection of Aβ42 within the same test sample, which was 18.7 pg/mL when the dilution factor was corrected (Supplementary Table 5), which cannot be achieved by relative quantification. As MSD recommend a minimum plasma dilution factor of 4-fold for use with their kits, the Aβ42 concentration was determined by the conventional method of relative quantification using the 4-fold diluted plasma giving a measurement of 4.86 ± 3.14 pg/mL albeit the LLOQ of the kit is 2.5 pg/mL. Hence the routine method of relative quantification gave rise to a result outside of the robust limits of quantification, with a measurement of 19.4 pg/mL Aβ42 within neat plasma following correction for the dilution factor. Nonetheless, these values of Aβ42 derived by routine relative quantification and by standard additions are consistent with Aβ42 levels in normal human plasma cited in literature^13^. Within the replicate experiment, the concentration of Aβ40 within the pooled plasma was determined to be 52.1 pg/mL by relative quantification when the 4-fold diluted plasma was analysed and corrected for the dilution factor, which is consistent with earlier findings in the present study of overestimation of Aβ40 content by relative quantification with this test plasma.

## Discussion

Using our standard additions approach, the test samples are spiked with known concentrations of calibrants such that the total concentration lies within the linear range of the dose-response curve. This requires an initial estimate of the endogenous concentration, which is subsequently refined as follows: using the fact that the log total concentration is close to linear with respect to the signal output, the initial estimate for the endogenous concentration is adjusted until a plot of log total concentration against signal response is indeed linear, thus indicating that the estimate is close to the true value. This can be achieved in a number of ways; in a later section of this paper we describe one method based on linear regression. Our method is easily implemented by the laboratory scientist using spreadsheet software such as Microsoft Excel. To illustrate this we provide an example as Supplementary File 1, we have produced an example spreadsheet which includes example data and shows how the solution is found. Readers may insert their own data into the file and use it in its current form or modify the spreadsheet as required.

From this study, there are key points to follow when implementing and validating the standard additions method of deriving endogenous analyte concentrations within biological samples for every new immunoassay as listed: (1) Knowledge of the conventional calibration curve in diluent and the physiological concentration range of the analyte are necessary to aid selection of the range and concentration of spike solutions to supplement the test sample to generate a linear correlation on a log-log plot. (2) Incorporate as many spike concentrations as possible so that the upper and lower asymptotes of the sigmoid may also be captured to help identify the maximum linear range as a smaller standard error corresponds to a better representation of the relationship between the signal response and the concentration. (3) The curve-fitting approach using standard additions is then applied to all the signal output data to establish the estimated endogenous analyte concentration. A data point is then omitted from one end, and the estimated endogenous analyte concentration and the line refitted is reassessed visually on a log-log plot. Data points that deviate from the linear trend on the log-log plot are sequentially discarded to the linear fit until the best solution is obtained for a minimum of four spike concentrations that may be suitable to use for other samples of the same matrix and dilution factor. For the immunoassays evaluated in this paper four spike concentrations gave the optimum result by balancing the number of points required to generate a robust linear trendline while offsetting greater technical variability resulting from the inclusion of more data points. (4) Confidence in identifying the longest linear portion is gained by assessing three of the four data points that define the longest linear range plus another neighbouring point outside the maximum linear range, and looking for an increase in the residual sum of squares in the resultant straight line when compared with using the four optimum points. If the four points selected encompass only a narrow portion of the linear range, erroneous results may be generated that lack supporting data. (5) When validating the approach, the acceptance criteria is concordance in endogenous analyte concentration within two or more distinct dilution factors of the same test samples when sample dilution is corrected. A variety of test samples entailing different concentrations of the endogenous analyte of interest should be assessed at more than one dilution factor to ascertain the suitability of four specific spike concentrations to be used for a given assay, with a specified analyte range and a defined matrix. While this approach requires multiple sampling of the test sample, it addresses differential matrix effects between true complex biological matrices and calibrant diluent. Despite the additional work required to initially determine the lowest suitable number of spike concentrations for a given sample type (i.e. a defined matrix of a particular dilution factor), and the need to establish sample linearity using more than one dilution factor when initially validating the approach, the standard additions method offers better quality and hence more meaningful measurements for disease-state diagnosis or for the validation of disease-state biomarkers. By eliminating the traditional standard curve, this approach is amenable for developing new portable POC devices with a restricted number of reaction chambers.

This technique is not limited to use for immunoassays but is a generic approach that is applicable to any data that produces a signal response (whether it be linear or log transformed) that necessitates log transformation of the analyte concentration to generate a linear trendline that may even reside within a sigmoidal plot.

## Methods

### Cortisol MSD Immunoassay

To evaluate the implementation of the standard additions method for the determining the concentration of total cortisol in serum, two distinct pooled human sera (one male pooled serum and one female pooled serum) that are certified reference materials (CRMs) were sourced from the National Institute of Standards and Technology (NIST). The endogenous cortisol levels of each pooled serum sample have been value assigned by LC-MS. The LC-MS determined concentrations of total cortisol within the neat pooled human NIST sera were 102.469 ng/mL and 86.417 ng/mL, respectively for the male and female NIST sera. The immunoassay used to measure total cortisol was developed in-house and is proprietary to LGC. The assay was performed using the Meso Scale SECTOR Imager 6000 instrument.

The Multi-Array High Bind 96-well microtiter plate (MSD) was coated with a mouse anti-cortisol monoclonal antibody (CalBioreagents, USA) overnight at 4 °C. The excess antibody was removed by inverting the plate, and then the plate was washed three times with PBS, 0.05% Tween20 wash buffer. Blocking buffer, comprising of 1% BSA in PBS, was added to each reaction well to saturate the vacant binding sites and the plate was incubated at RT for 1 h with shaking at 450 rpm. The plate was then emptied by inversion, and rinsed thrice with the wash buffer. Cortisol spike solutions were prepared to the following concentrations: 200, 150, 112.5, 84.38, 63.28, 47.46, 35.60, 26.70, 20.02, 15.02, 11.27 and 0 ng/mL cortisol using a CRM from NIST (NIST 921) which were termed as spike solutions 1–12, respectively, using a 1.333 fold serial dilution from 200 ng/mL cortisol in a phosphate buffer, pH 7.2 The male and female NIST sera (NIST 971) were separately diluted 6-fold in a phosphate buffer, pH 7.2. The 6-fold diluted test sera were each effectively diluted a further 2-fold, following the equivolume addition of the cortisol spike solutions, such that the resultant spike quantities were equivalent to 100, 75, 56.25, 42.19, 31.64, 23.73, 17.80, 13.35, 10.01, 7.51, 5.63, and 0 ng exogenous cortisol within 1 mL of spiked serum mixture.

This 12-fold diluted test serum (with or without additional exogenous spiked cortisol) was then supplemented with an equivolume of tracer, cortisol-HRP (Calbioreagents, USA), in a phosphate-based buffer with reagents that dissipate the binding of cortisol to serum proteins, prior to addition of 50 μL of the resultant mixture to the wells of the microtiter plate. The reaction mixture was incubated for 1 h at RT with agitation of the plate at 450 rpm. The plate contents were then decanted by inversion, before the plate was rinsed three times with wash buffer prior to the addition of the biotinylated anti-HRP detection antibody (25 μL per well). The plate was subsequently incubated for 1 h at RT with agitation. The plate was then emptied by inversion, and rinsed thrice with the wash buffer before the addition of MSD SULFO-TAG conjugated with streptavidin (25 μL per well), for 1 h at RT with shaking. The contents were decanted by inversion, before the plate was rinsed three times with wash buffer before addition of MSD 1x Read Buffer T with surfactant. The plate was loaded onto the MSD SECTOR Imager 6000 instrument, and a voltage is applied to the electrodes integrated within the base of the microtiter plate to initiate electroluminescence. The ruthenium within the MSD SULFO TAG drives a redox reaction with the tripropylamine in the MSD Read Buffer T with surfactant and emits light that is detected by the cooled CCD camera within the imager, and the MSD Workbench v3.0 software was used for the data analysis.

### Aβ40 and Aβ42 MSD Immunoassays

The MSD V-PLEX Aβ Peptide Panel 1 (4G8) kit was used, as per the manufacturer’s instructions, except the test samples were spiked as described. Spike solutions 1–12 were prepared from the kit calibrants in Diluent 35 to contain 14900, 7450, 3725, 1862.5, 931.25, 465.63, 232.81, 116.41, 58.20, 29.10, 14.55 and 0 pg/mL Aβ40, and also 1730, 865, 432.5, 216.25, 108.13, 54.06, 27.03, 13.52, 6.76, 3.34, 1.69, 0.84, 0.42 pg/mL Aβ42, respectively. The test sera, pooled female plasma and individual female plasma termed A07, were sourced from Sera Laboratories UK. The test plasma (either 2-fold and 6-fold diluted for the first experiment, or neat and 2-fold diluted for the second experiment) was supplemented with an equivolume of exogenous calibrants in the forms of spike solutions 1–12 such that their net exogenous Aβ concentrations were halved. Spike solutions 1, 3, 5, 7, 9, 11 and 13, which correlate to the calibrants recommended for use in the kit, were used as the calibrants for relative quantification. Following the addition of detection antibody to the plate, 25 μL of each calibrant and each diluted plasma test sample (±exogenous Aβ peptides) was added to the plate with duplicate loadings at RT for 2 h with shaking, before washing and the addition of the Read Buffer T. The plate was then analysed using the MSD SECTOR Imager 6000.

### Implementing The Method To Estimate The Endogenous Analyte Concentration Using Linear Regression To Plot The Immunoassay Signal Output

The method we have used consists of a linear regression of log Y on log (S + U), where Y is the observed signal response, S is a spiking concentration and U is the unknown endogenous concentration. It is thus assumed that the linear portion of the sigmoidal response is adequately approximated by a linear relationship. Given that the values of Y and S are known, determining the value of U which produces the lowest deviation from linearity is achieved with a numerical method which minimises or maximises a suitable linearity parameter. For example, one might fit a quadratic regression for each value of U, then accept the solution whose quadratic term is closest to zero. Alternatively and more simply, a linear regression can be used instead, and the best solution deemed to be that which has the smallest residual variation. This is the method we have used in this work. Other approaches are also possible, and it may well be the case that with additional study better performing methods can be found. However, the simple linear regression approach is fit for the purpose outlined in this paper, and serves well as an illustration. We have constructed a simple spreadsheet using Microsoft Excel (available for download as Supplementary File 1) which uses the Solver function to minimise the residual variation and find the best fit endogenous concentration (see Supplementary Fig. 2).

We investigated the performance of the method over many repeated experiments by simulation. For example, in one such exercise, the true endogenous concentration was set to 30 ng/mL with spike concentrations of 25, 50, 100 and 200 ng/mL and a defined measurement repeatability standard deviation. Using a fixed underlying linear relationship between log total concentration and log signal response, simulated data sets were generated and our method applied to each data set. Supplementary Fig. 4 shows two sets of results for 10,000 replicate data sets and two repeatability standard deviations. The distribution is slightly asymmetric, which introduces a small bias as the mean is higher than the median value of 30 ng/mL. The median is 30 ng/mL as expected, since half the estimates are above the true value, and half are below. However, this bias is small compared to the variability of the estimates, although it increases as the measurement repeatability worsens. Thus, our method will perform best when the measurement precision is good, and there are no additional sources of uncertainty, such as dilution errors.

The simulation provides an indication of the uncertainty associated with the estimate, and can be applied to a single estimate obtained from a single set of data. Since the residual distribution is known to be approximately normal and constant, and we have observed signal response values for each spike level, we can use a parametric bootstrap to carry out the simulation described above and obtain an approximate confidence interval on the result. The procedure is as follows: calculate the repeatability standard deviation from the data, then use this and the mean for each spike level to generate bootstrap data sets by drawing from the appropriate normal distributions. For each data set obtain the estimated endogenous concentration to obtain the distribution of bootstrap estimates. The 2.5% and 97.5% quantiles of this distribution are used as approximate 95% confidence limits. A worked example is given in Supplementary Fig. 5.

## Electronic supplementary material

Supplementary Information

Dataset 1
