# Supplementary material for: A generic standard additions based method to determine endogenous analyte concentrations by immunoassays to overcome complex biological matrix interference
Source: Sci Rep. 2017 Dec 13;7:17542. doi: 10.1038/s41598-017-17823-y (PMC5727527; doi:10.1038/s41598-017-17823-y)
Supplement: Supplementary file 1 — Supplementary Information [file 41598_2017_17823_MOESM1_ESM.pdf]

A generic standard additions based method to determine endogenous analyte concentrations by immunoassays to overcome complex biological matrix interference

Susan Pang[1] and Simon Cowen[1]

[1]LGC, Queens Road, Teddington, UK

Correspondence should be addressed to S.P. (Susan.Pang@lgcgroup.com)

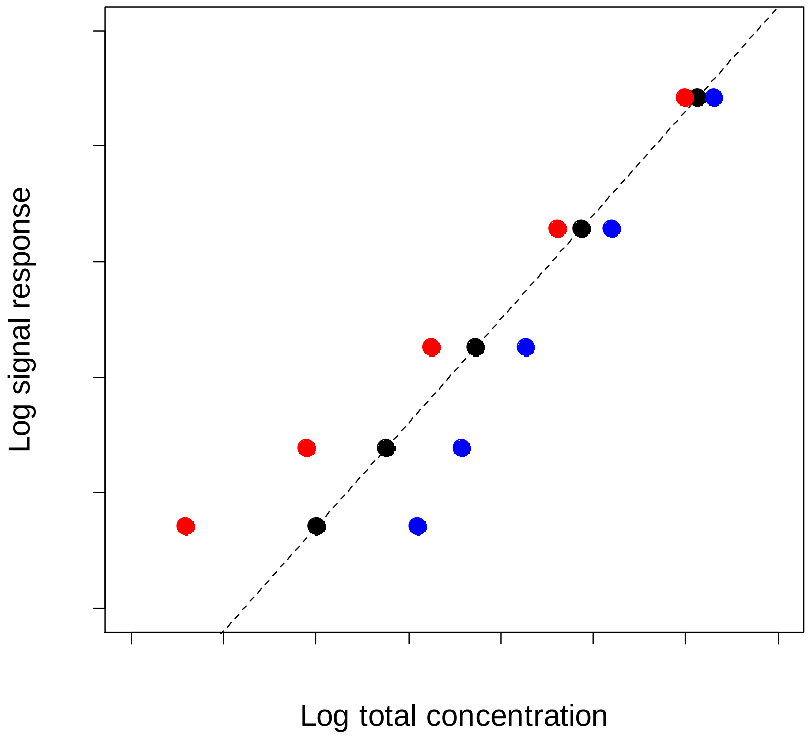

Supplementary Fig. 1. Illustrations depicting the expected relationship between the estimated total concentration and the observed log response. Here the estimated endogenous concentration is equal to (black), below (red) or above (blue) the true value. A linear response indicates that the estimate is equal to the true value.

.

Test value

U = 9.395

Data

Spike conc S	Signal response Y	ln(Y)	ln(S+U)
100.00	228.00	5.4293	4.6950
75.00	282.67	5.6443	4.4355
56.25	343.33	5.8387	4.1843
42.19	426.33	6.0552	3.9432
31.64	531.67	6.2760	3.7144
23.73	638.00	6.4583	3.5003
17.80	769.67	6.6460	3.3029
13.35	904.67	6.8076	3.1243
10.01	1067.67	6.9732	2.9656
7.51	1181.00	7.0741	2.8275
5.63	1313.00	7.1801	2.7098
0.00	1956.00	7.5787	2.2401

Regression

	x	x^2	y	xy	Predicted y	Residual	Residual^2
	4.69496	22.04267	5.42935	25.49057	5.40671	0.02264	5.124E-04
	4.43550	19.67370	5.64427	25.03517	5.63764	0.00663	4.390E-05
	4.18426	17.50800	5.83870	24.43062	5.86127	-0.02257	5.093E-04
	3.94318	15.54863	6.05522	23.87680	6.07584	-0.02062	4.253E-04
	3.71443	13.79701	6.27602	23.31184	6.27944	-0.00342	1.172E-05
	3.50029	12.25204	6.45834	22.60607	6.47004	-0.01170	1.369E-04
	3.30294	10.90942	6.64596	21.95121	6.64569	0.00026	7.011E-08
	3.12426	9.76100	6.80757	21.26860	6.80473	0.00284	8.041E-06
	2.96558	8.79466	6.97323	20.67967	6.94597	0.02727	7.434E-04
	2.82750	7.99475	7.07412	20.00205	7.06887	0.00525	2.757E-05
	2.70978	7.34292	7.18007	19.45643	7.17364	0.00643	4.133E-05
	2.24014	5.01823	7.57866	16.97726	7.59165	-0.01299	1.688E-04
sum	41.64282	150.64302	77.96149	265.08629			2.629E-03

Plot

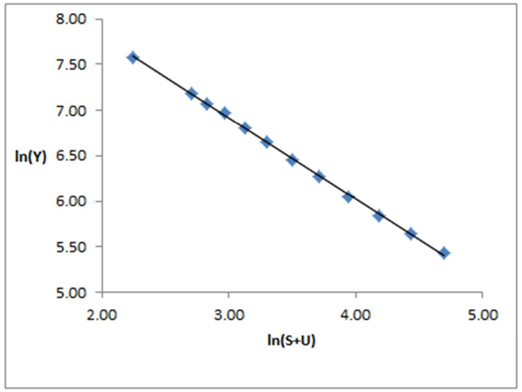

Gradient	-0.8901
Intercept	9.5855
n	12
Residual sum of squares	2.629E-03

Instructions for use

1. Put data into the grey shaded area in columns B and C without log transforming first
2. Change the value in cell C3 until the plot looks approximately linear. This is your initial estimate
3. Press the button below to run the Excel Solver and find the best solution

Find best fit U

Supplementary Fig. 2 Backfitted linear regression derived concentration for the 12-fold male (this page) and female (next page) NIST sera.

In each case the estimate of the unknown endogenous concentration U is varied until a regression of natural log response against natural log total cortisol produces a fitted line with minimum residual variation.

Test value

U = 7.710

Data

Spike conc S	Signal response Y	ln(Y)	ln(S+U)
100.00	222.33	5.4042	4.6794
75.00	284.67	5.6513	4.4153
56.25	358.33	5.8815	4.1583
42.19	442.67	6.0928	3.9100
31.64	518.67	6.2513	3.6725
23.73	648.67	6.4749	3.4481
17.80	785.67	6.6665	3.2390
13.35	899.00	6.8013	3.0473
10.01	997.33	6.9051	2.8747
7.51	1149.00	7.0466	2.7225
5.63	1323.33	7.1879	2.5908
0.00	2103.00	7.6511	2.0425

Regression

	x	x^2	y	xy	Predicted y	Residual	$Residual^2$
	4.67944	21.89715	5.40418	25.28852	5.42714	-0.02297	5.274E-04
	4.41534	19.49519	5.65132	24.95247	5.64886	0.00246	6.050E-06
	4.15825	17.29106	5.88146	24.45661	5.86468	0.01678	2.816E-04
	3.90996	15.28781	6.09282	23.82269	6.07312	0.01970	3.879E-04
	3.67250	13.48727	6.25126	22.95777	6.27247	-0.02121	4.498E-04
	3.44808	11.88928	6.47492	22.32606	6.46087	0.01405	1.974E-04
	3.23897	10.49094	6.66653	21.59271	6.63642	0.03011	9.067E-04
	3.04728	9.28592	6.80128	20.72542	6.79735	0.00394	1.550E-05
	2.87475	8.26416	6.90509	19.85036	6.94219	-0.03710	1.377E-03
	2.72249	7.41193	7.04665	19.18439	7.07001	-0.02337	5.460E-04
	2.59084	6.71245	7.18791	18.62273	7.18053	0.00738	5.446E-05
	2.04247	4.17168	7.65112	15.62718	7.64089	0.01023	1.047E-04
sum	40.80037	145.68486	78.01453	259.40693			4.854E-03

Plot

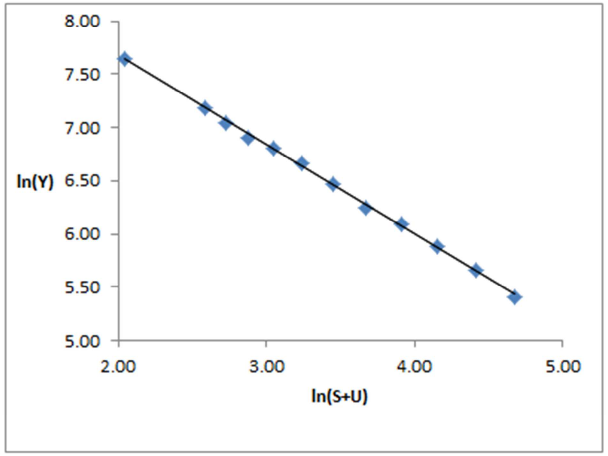

Gradient	-0.8395
Intercept	9.3555
n	12
Residual sum of squares	4.854E-03

Instructions for use

1. Put data into the grey shaded area in columns B and C without log transforming first
2. Change the value in cell C3 until the plot looks approximately linear. This is your initial estimate
3. Press the button below to run the Excel Solver and find the best solution

Find best fit U

Replicate	Sample ID	Net Spike Concentration within 12-fold diluted test serum (ng/mL)	Expt 1 Signal Output (AU)	Expt 2 Signal Output (AU)	Expt 3 Signal Output (AU)
1	M1	50	370	387	704
2	M1	50	394	395	740
3	M1	50	390	423	779
1	M2	25	607	657	1223
2	M2	25	628	695	1272
3	M2	25	609	714	1292
1	M3	12.5	973	1046	1972
2	M3	12.5	966	1082	1769
3	M3	12.5	940	1040	1926
1	M4	0	1879	1947	3841
2	M4	0	1911	2071	3905
3	M4	0	1948	2102	3866
1	F1	50	336	414	800
2	F1	50	327	396	765
3	F1	50	306	362	722
1	F2	25	594	661	1295
2	F2	25	548	688	1295
3	F2	25	610	637	1188
1	F3	12.5	887	1092	1983
2	F3	12.5	919	1063	1981
3	F3	12.5	825	1020	1890
1	F4	0	1959	2276	4283
2	F4	0	2010	2376	4411
3	F4	0	1988	2126	4171

Supplementary Table 1. Signal output data from male and female NIST sera spiked with NIST cortisol standards. Each serum was supplemented with cortisol standards such that the net exogenous cortisol was 0, 12.5, 25 and 50 ng within 1 mL of the resultant test sample of 12-fold diluted NIST sera. The 6-fold diluted NIST sera were supplemented with an equivolume of 1, 25, 50 and 100 ng/mL cortisol standards to give samples M1, M2, M2 and M4, respectively. The female NIST serum from spiked in the same manner to produce F1, F2, F3 and F4 samples with the net exogenous cortisol quantities of 0, 12.5, 25 and 50 ng within 1 mL. The cortisol assays were performed using the MSD platform and the signal outputs from determinations of the each sample from three separate experiments are tabulated.

<table>
<tr><th colspan="3"></th><th colspan="4">Standard additions derived data determined using linear regression</th><th colspan="4">Conventional relative quantification data derived by interpolation from an internal standard curve</th></tr>
<tr><th>Expt #</th><th>Format of data used for linear fit of the sequence of 4 points with net exogenous concentrations of (0, 12.5, 25, 50 ng/mL)</th><th>NIST serum 971</th><th>[Cortisol] (ng/mL)</th><th>Mean [Cortisol] (ng/mL)</th><th>SD</th><th>CV (%)</th><th>[Cortisol] (ng/mL)</th><th>Mean [Cortisol] (ng/mL)</th><th>SD</th><th>CV (%)</th></tr>
<tr><td>1</td><td>Mean of triplicate determinations</td><td>Male</td><td>136.26</td><td rowspan="3">158.85</td><td rowspan="3">28.53</td><td rowspan="3">18.0</td><td>115.14</td><td rowspan="3">121.42</td><td rowspan="3">5.51</td><td rowspan="3">4.5</td></tr>
<tr><td>2</td><td>Mean of triplicate determinations</td><td>Male</td><td>190.91</td><td>123.64</td></tr>
<tr><td>3</td><td>Mean of triplicate determinations</td><td>Male</td><td>149.37</td><td>125.46</td></tr>
<tr><td>1</td><td>Mean of triplicate determinations</td><td>Female</td><td>134.98</td><td rowspan="3">128.71</td><td rowspan="3">13.77</td><td rowspan="3">10.7</td><td>108.67</td><td rowspan="3">107.71</td><td rowspan="3">1.19</td><td rowspan="3">1.1</td></tr>
<tr><td>2</td><td>Mean of triplicate determinations</td><td>Female</td><td>138.23</td><td>106.39</td></tr>
<tr><td>3</td><td>Mean of triplicate determinations</td><td>Female</td><td>112.92</td><td>108.08</td></tr>
<tr><td>3</td><td>Single determination: 1st replicate only</td><td>Male</td><td>202.14</td><td rowspan="3">152.38</td><td rowspan="3">46.35</td><td rowspan="3">30.4</td><td colspan="4" rowspan="6"></td></tr>
<tr><td>3</td><td>Single determination: 2nd replicate only</td><td>Male</td><td>110.45</td></tr>
<tr><td>3</td><td>Single determination: 3rd replicate only</td><td>Male</td><td>144.54</td></tr>
<tr><td>3</td><td>Single determination: 1st replicate only</td><td>Female</td><td>108.97</td><td rowspan="3">112.98</td><td rowspan="3">3.65</td><td rowspan="3">3.2</td></tr>
<tr><td>3</td><td>Single determination: 2nd replicate only</td><td>Female</td><td>116.11</td></tr>
<tr><td>3</td><td>Single determination: 3rd replicate only</td><td>Female</td><td>113.86</td></tr>
</table>

Supplementary Table 2. Comparison of the linear regression derived endogenous cortisol concentration with different replicate determinations. The concentrations of cortisol were derived using the standard additions method for male and female NIST serum (971) using the mean of triplicate determinations or single determinations of one unspiked and three spiked samples for each test sample in three separate experiments. In this instance the three spike concentrations were used to emulate coverage of the upper physiological range of human serum cortisol rather than for the optimum spike concentrations to ascertain the endogenous concentration, and the data enable assessment of the reproducibility of the method. The right four columns shows data derived using relative quantification whereby the signal correlating to the unspiked male or female NIST sera were interpolated from the internal calibration curve.

Points	Net plasma dilution after spike addition	Plasma	WS spikes used in linear regression method	Linear regression derived concentration of Aβ40 in diluted pooled plasma (pg/mL)	Residual sum of squares	Aβ40 in equivalent neat plasma from optimum linear range (pg/mL)
4	4	Pooled	7-10	33.02	0.002064	
3	4	Pooled	7-9	14.72	2.51E-26	
4	4	Pooled	6-9	10.27	0.000234	= 41.1
3	4	Pooled	6-8	3.65	4.54E-28	
9	4	Pooled	2-10	4.22	0.109255	
8	4	Pooled	3-10	7.43	0.052595	
8	12	Pooled	4-11	3.77	0.038705	
7	12	Pooled	5-11	5.46	0.011297	
6	12	Pooled	6-11	6.74	0.005848	
5	12	Pooled	7-11	7.13	0.005658	
4	12	Pooled	7-10	3.71	0.003346	= 44.5
3	12	Pooled	7-9	20.42	1.27E-18	
5	12	Pooled	5-9	4.26	0.00542	
6	12	Pooled	5-10	3.13	0.005664	
10	4	A07	1-10	2.89	0.324603	
9	4	A07	2-10	8.29	0.092678	
8	4	A07	3-10	12.93	0.028746	
7	4	A07	4-10	17.02	0.009726	
6	4	A07	5-10	18.85	0.008277	
5	4	A07	6-10	27.37	1.59E-05	
4	4	A07	7-10	27.31	1.59E-05	
4	4	A07	6-9	26.79	8.91E-06	
3	4	A07	7-9	25.27	1.3E-26	= 107.2
3	4	A07	6-8	28.69	2.69E-18	
4	4	A07	5-8	1.79	0.003294	
8	12	A07	4-11	7.58	0.021569	
7	12	A07	5-11	9.52	0.00566	
6	12	A07	6-11	9.14	0.005399	
5	12	A07	7-11	11.62	0.001774	
4	12	A07	8-11	10.51	0.001599	
3	12	A07	8-10	3.58	4.93E-28	
4	12	A07	7-10	9.81	0.001409	= 117.7

Supplementary Table 3. Elucidation of the endogenous Aβ40 concentration in diluted pooled plasma and individual plasma A07. The linearity from the inclusion of different data points in the linear regression was assessed in order to obtain the points depicting the maximum linearity. The resultant endogenous Aβ40 concentrations derived for each plasma at each dilution from the combination of four data points with the smallest residual sum of squares (and hence maximum linearity) is highlighted for each plasma at each dilution factor.

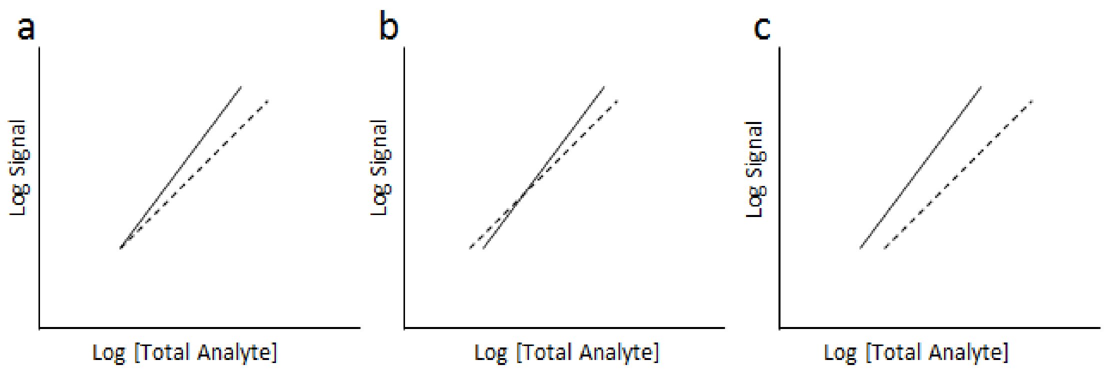

Supplementary Fig 3. Scenarios depicting the possible localisation of the linear portion of the calibration curve and spiked test samples when the test samples exhibit more profound matrix effects due to increased complexity compared with calibrant diluent. Of the three possible scenarios, panel a is the theoretical option, whereas the most likely experimental possibilities are depicted by panels b and c.

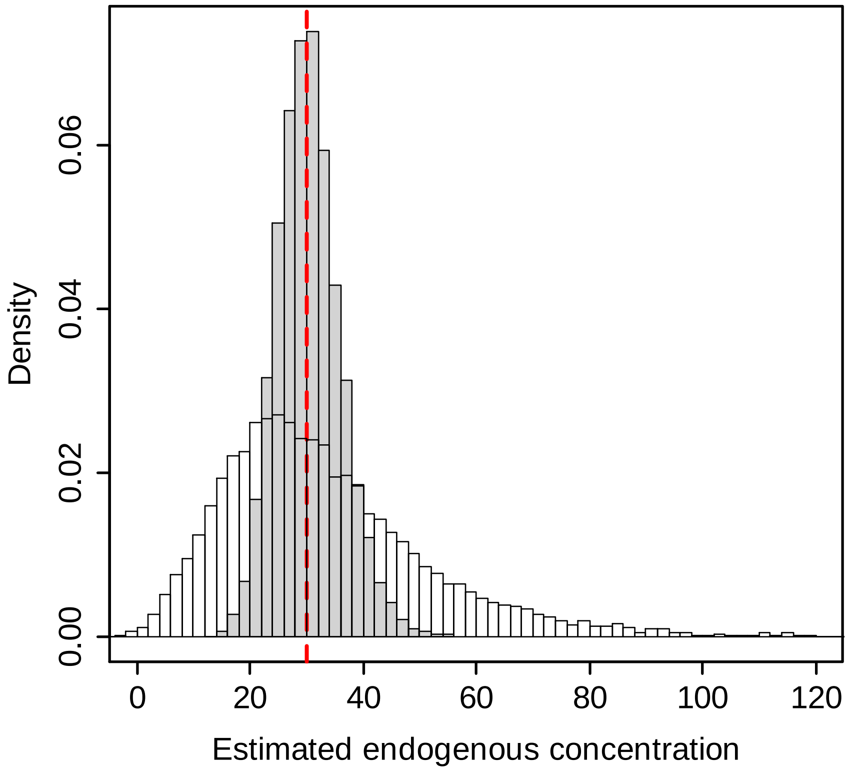

Supplementary Fig. 4. Distribution of the estimates of the endogenous concentration obtained from a simulation exercise. Two repeatability levels are shown: a standard deviation of 0.01 on the log response scale (grey) and 0.02 (white). In each case, a known concentration of 30 ng/mL and a defined underlying sigmoidal relationship were used to create 10,000 data sets to which our estimation method was then applied. The median remains constant at 30 ng/mL, but the mean increases as the measurement precision worsens.

Points	Dilution factor	Plasma	Spike calibrants used in linear regression method	Linear regression derived concentration of Aβ40 in diluted pooled plasma (pg/mL)	Residual sum of squares	Aβ40 in equivalent neat plasma from optimum linear range (pg/mL)
8	4	Pooled	3-10	7.48	0.023474	= 40.2
7	4	Pooled	4-10	10.28	0.0007388	
6	4	Pooled	4-9	9.00	0.007037	
5	4	Pooled	4-8	7.02	0.006873	
5	4	Pooled	5-9	14.46	0.001871	
4	4	Pooled	5-8	23.72	0.000762	
4	4	Pooled	4,6,8,10	10.04	3.95xE-07	
4	4	Pooled	3,5,7,9	2.63	0.015363	
4	4	Pooled	3,5,8,10	6.18	0.018461	
4	4	Pooled	4-7	-7.31	0.00555	
4	4	Pooled	4,6,8,9	9.14	1.63xE-05	
11	2	Pooled	2-12	21.77	0.15786	= 40.2
7	2	Pooled	5-11	34.55	0.008123	
6	2	Pooled	6-11	40.07	0.005726	
6	2	Pooled	4-9	20.32	0.000248	
5	2	Pooled	7-11	54.78	0.003274	
4	2	Pooled	5,7,9,11	33.82	0.005895	
4	2	Pooled	4,6,8,10	31.52	0.00279	
4	2	Pooled	7-10	68.38	0.002413	
4	2	Pooled	8-11	75.50	0.003035	
4	2	Pooled	4,6,8,9	20.89	0.000155	
4	2	Pooled	5,6,7,8	17.96	2.69xE-05	
4	2	Pooled	5-9	21.34	0.000156	
4	2	Pooled	4,5,7,9	19.89	2.69xE-05	
4	2	Pooled	4,6,7,9	20.08	5.64xE-08	

Supplementary Table 4. Deriving endogenous Aβ40 concentrations in plasma by linear regression. Linear regression derived Aβ40 in pooled female plasma, with a net dilution of 2-fold or 4-fold, following the supplementation with the spike solutions comprising of Aβ40 calibrants. Note that in this particular assay, the temperature of the assay was approximately 3 ^{o}C higher at 25.8 ^{o}C compared with the first experiment (data reported in Supplementary Table 3 and Table 3), and the speed of the plate shaker was increased from 450 rpm to 750 rpm. The endogenous Aβ40 concentration with the smallest residual sum of squares is highlighted for each dilution factor.

Spike (ng/mL)	Total (ng/mL)	Log total conc	Signal response			Log signal response			Mean log response
50.0	61.4	1.788	370	394	390	5.9135	5.9764	5.9661	5.9520
25.0	36.4	1.561	607	628	609	6.4085	6.4425	6.4118	6.4210
12.5	23.9	1.378	973	966	940	6.8804	6.8732	6.8459	6.8665
0.0	11.4	1.057	1879	1911	1948	7.5385	7.5554	7.5746	7.5561

Endogenous concentration
Mean bootstrap estimate 11.40 ng/mL

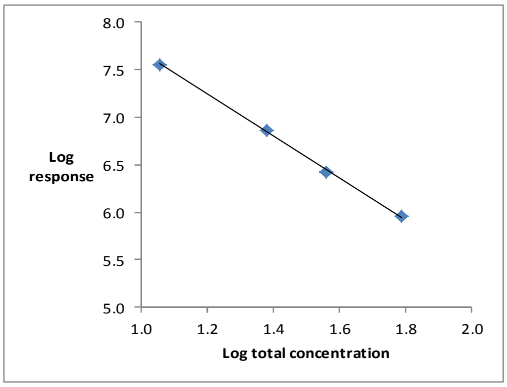

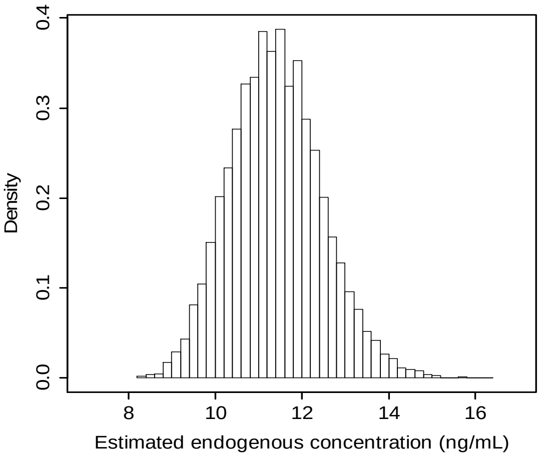

95% confidence interval: 9.4 to 13.6 ng/mL

Supplementary Fig. 5. Uncertainty estimate obtained by bootstrapping. The data set consists of the cortisol assay and has a best fit endogenous concentration of approximately 8.3 ng/mL. The repeatability standard deviation of the log response was estimated to be 0.046 from the individual measurements. Using this and the mean log response for each spike level as the parameters for four normal distributions (one for each spike level), 10,000 data sets were sampled and the corresponding estimates obtained. An approximate 95% confidence interval of [9.4, 13.6] ng/mL was then obtained from the resulting distribution.

Points	Dilution factor	Plasma	Calibrant spikes used in linear regression method	Linear regression derived concentration of Aβ42 in diluted pooled plasma (pg/mL)	Residual sum of squares	Aβ42 in equivalent neat plasma from optimum linear range (pg/mL)
11	2	Pooled	2-12	10.52	0.045334	
8	2	Pooled	5-12	17.38	0.00867	
7	2	Pooled	2-8	6.83	0.012901	
6	2	Pooled	5-10	20.65	0.004164	
6	2	Pooled	3-8	8.49	0.0005034	
5	2	Pooled	2-6	0.71	0.004094	
5	2	Pooled	8-12	-10.43	N/A	
5	2	Pooled	4-8	8.72	0.004984	
5	2	Pooled	5-9	17.24	0.001192	
5	2	Pooled	3-7	6.80	0.003614	
4	2	Pooled	2,4,6,8	7.56	0.002747	
4	2	Pooled	2,5,8,11	9.22	0.019336	
4	2	Pooled	4,6,8,10	14.72	0.007903	
4	2	Pooled	4,6,8,9	12.23	0.002757	
4	2	Pooled	4,6,7,8	10.03	4.15xE-05	
4	2	Pooled	4,6,7,9	12.63	0.000729	
4	2	Pooled	4,5,7,8	7.44	0.00352	
4	2	Pooled	4,5,7,9	11.12	0.007876	
4	2	Pooled	4,5,8,9	9.64	0.008418	
4	2	Pooled	4,5,6,9	10.74	0.005611	
4	2	Pooled	5,6,8,9	18.12	0.001044	
4	2	Pooled	3,4,6,7	9.33	2.47xE-08	= 18.7
4	2	Pooled	3,5,6,7	6.39	0.002598	
4	2	Pooled	3,4,5,7	5.92	0.001943	
4	2	Pooled	3,5,7,8	7.55	0.002577	
4	2	Pooled	3,4,7,8	9.97	5.59xE-05	
4	2	Pooled	3,6,7,8	9.90	4.8xE-05	
4	2	Pooled	3,5,6,8	8.33	0.003855	

Supplementary Table 5. Linear regression derived Aβ42 in pooled female plasma. Following the supplementation of neat plasma with an equivolume of the spike solutions comprising of Aβ42 calibrants, the plasma has a net dilution of 2-fold. The data points comprising of the plasma with Spike solutions 3, 4, 6 and 7 gave rise to an endogenous Aβ42 concentration of 18.7 pg/mL within the plasma when corrected for the dilution factor, with the smallest residual sum of squares observed for any given combination of four datapoints. There is concordance with this concentration when linear regression is used to derive the concentration of Aβ42 from plasma spiked with calibrants 4,6,7,8 (20.0 pg/mL), calibrants 3,6,7,8 (19.8 pg/mL) and calibrants 3,4,7,8 (19.9 pg/mL), each with residual sum of squares of ≤ 5.59xE-05.
